# Assessment of cancer biomarkers in the Grenfell firefighter cohort study

**DOI:** 10.1038/s41598-025-95991-y

**Published:** 2025-05-07

**Authors:** Johanna Feary, Yizhou Yu, Tamanna Kabir, Susie Schofield, Adrian Bevan, Victoria Askinyte, Katherine Honan, Liza Emirali, Andrea Rubbi, Anne E. Willis, Paul Cullinan, Shubha Anand, L. Miguel Martins

**Affiliations:** 1 National Heart & Lung Institute, Imperial College, Emmanuel Kaye Building 1B, Manresa Road, London, SW3 6LR UK; 2 MRC Toxicology Unit, University of Cambridge, Gleeson Building, Tennis Court Road, Cambridge, CB2 1QR UK; 3 Cancer Molecular Diagnostics Laboratory, Department of Oncology, University of Cambridge, The Clifford Allbutt Building, Cambridge Biomedical Campus, Hills Road, Cambridge, CB2 0AH UK; 4 Royal Brompton Hospital, Guys and St Thomas’ NHS Foundation Trust, London, SW3 6LR UK; 5 London Fire Brigade, Union Street, London, UK SE1 0LL; 6 London, UK

**Keywords:** Firefighter, Fire smoke, Cancer, Biomarkers, Biomarkers, Diagnostic markers, Cancer epidemiology, Natural hazards

## Abstract

Firefighters are exposed to a diverse range of harmful substances, including polycyclic aromatic hydrocarbons, benzene, and other carcinogens. These toxic compounds induce DNA damage, often causing the formation of DNA adducts and other lesions that can contribute to the development of various diseases, including cancer. Recent advancements in molecular diagnostics have shown that circulating cell-free DNA (cfDNA) in plasma is a valuable biomarker for detecting DNA damage and disease states. In this study, we explored whether changes in the quantity and quality of plasma cfDNA might reveal DNA lesions or serve as early markers for diseases such as cancer in UK firefighters. Whilst there are few published epidemiological studies of risk of cancer in UK firefighters, there are none on molecular markers in this population. All the 685 firefighters who participated in the study were employed by the London Fire Brigade in 2017; many of them also attended the Grenfell Tower fire, the most devastating fire to occur in the UK in modern history. In this exploratory analysis, we sought to gain insights into the potential long-term health impacts of toxic smoke exposure on these first responders by analysing both the concentration of cfDNA present and specific genetic alterations in cfDNA. Using next-generation sequencing and a panel that detects pathogenic DNA variants linked to various cancers, we analysed a subset of 261 firefighters. Our findings revealed that 11 firefighters carried pathogenic DNA variants associated with cancer, but we found no association between fire smoke exposure and the presence of these variants.

## Introduction

The global firefighter community comprises over 15 million individuals, including both paid professionals and dedicated volunteers. Firefighters are exposed to fire smoke in urban, rural and wildfire settings and to other hazardous substances during events such as building collapses. There has been increasing interest in the health of firefighters, especially in their risk of malignant disease. The relevant literature on this topic is broad, but the findings are inconsistent^1,2^. However, in 2023, the International Agency for Research on Cancer (IARC) upgraded the classification of occupational exposure as a firefighter to Group 1 on the basis that there is sufficient evidence in humans that such occupational exposure can cause mesothelioma (58% higher risk (95% confidence interval: 14–120%)) which is known to be associated with asbestos exposure and bladder cancer (16% higher risk (95% confidence interval 8–26%). They found limited evidence that firefighters have an increased risk of other types of cancer, specifically colon, prostate and testicular cancers, melanoma of the skin, and non-Hodgkin’s lymphoma^3^.

Although the spectrum of toxic substances produced by structural fires is wide and generally unpredictable, common components of fire smoke include established human carcinogens such as benzene, polycyclic aromatic hydrocarbons (PAHs), polychlorinated biphenyls, asbestos, arsenic, 1,3-butadiene, formaldehyde and cadmium^3^. Exposure to these compounds may occur via inhalation or through the skin, including through the wearing of contaminated clothing and protective equipment^4,5^. Firefighters exposures to fire smoke are intermittent and may be infrequent^3^ however, increases in the concentrations of PAHs and volatile organic compounds have been detected in the urine and exhalant of firefighters following very short exposures to smoke^6,7^.

Several short-term molecular studies of cancer risk in firefighters have been conducted with varying results^3^. A more recent study of 53 Danish recruits undergoing fire training^8^ reported an association between the number of DNA strand breaks with dermal exposure to pyrene and total PAHs and concluded that firefighting activity is associated with cellular genotoxicity. It is also accepted that certain environmental exposures are associated with long-term epigenetic changes ranging from alterations in DNA and RNA methylation patterns, to changes in the expression of small noncoding RNAs. For example, cigarette smoking leaves a signature of DNA methylation that is detectable in blood more than 20 years after smoking has ceased^9,10^; similarly, occupational exposure to ionising radiation results in a defined signature that is detectable for up to four years after a single exposure event^11^. There are no published studies of alterations in the epigenomes of firefighters following occupational smoke inhalation or exposure through dermal routes.

In eukaryotic cells, the majority of DNA is stored within the cell nucleus; smaller amounts are found in the mitochondria. ‘Cell-free DNA’ (cfDNA) consists of small fragments of DNA between 90 and 150 base pairs in length that freely circulate in the bloodstream and are released from both the nucleus and the mitochondria (mtDNA). CfDNA, arising primarily from the bone marrow and white blood cells, is present in the blood of all individuals^12,13^ and can indicate cell death and tissue damage. Levels of cfDNA increase with age (probably due to the increase in incident age-related disease)^14^ and after exercise^15^ and may also be elevated in individuals with certain acute or chronic diseases^16,17^. Environmental exposure may also be associated with elevated levels of cfDNA. For example, in people with smoke inhalation injury, serum cfDNA levels were found to be higher than those in healthy controls, and they were also higher in individuals who were admitted to the hospital due to smoke inhalation than in those who experienced smoke inhalation injury but did not require hospitalisation^16^. In occupational studies, elevated levels of cfDNA have also been observed in interventional cardiologists who have been exposed to radiation^18^, and higher levels of circulating cfDNA originating from mitochondria have been found in workers exposed to fumigants or pesticides than in unexposed controls^19^.

In addition to quantitative variations, there may be qualitative changes in cfDNA in base pairs coding for genes associated with cancer risk; specifically, oncogenes can cause the active development of cancer and tumour suppressor genes. Mutations in the cfDNA of healthy subjects may reflect the effect of ongoing exposure to environmental carcinogens, particularly in individuals with genetic polymorphisms, e.g., *TP53* mutations, that predispose them to increased levels of mutagenic damage (reviewed in^20^).

In June 2017, a fire occurred in Grenfell Tower in London, United Kingdom; it resulted in the death of 72 residents and was attended by over 1000 firefighters who were members of the London Fire Brigade (LFB)^21^. The degree of exposure to fire smoke during this fire was unusually high for some firefighters who experienced the fire for a prolonged duration of time and, in some cases, without the use of appropriate breathing apparatus. In 2019, LFB firefighters, including many who had attended the Grenfell Tower fire, were recruited to the Grenfell Firefighter Study (GFS) cohort^22^.

Here, we report an analysis of quantitative and qualitative changes in cfDNA in a UK population of firefighters. The aim of the study was to determine if this approach could identify cancer-specific variants in cfDNA. The study was carried out in the GFS cohort stratified by attendance at the Grenfell Tower fire and historical occupational fire smoke exposure. We hypothesised that exposure to higher levels of fire smoke toxicants would be associated with a higher prevalence of cfDNA variants compared to those with lower exposures. An analysis of this kind has not been reported in a firefighter population to date.

## Results

### Analysis of cfDNA levels in a subset of the Grenfell Firefighters cohort

**Fig. 1:**
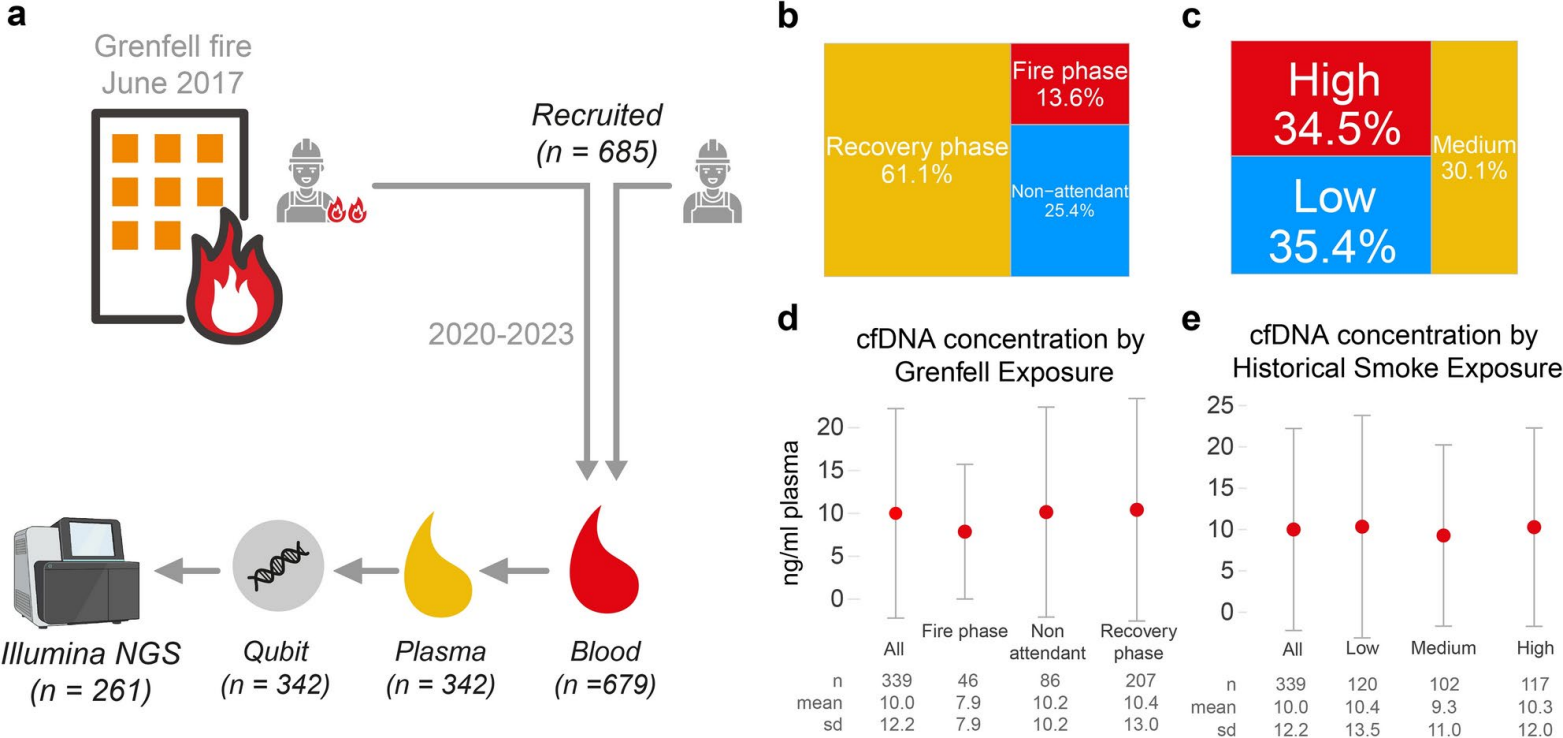
Analysis of cfDNA in the blood of firefighters belonging to the London Fire Brigade. (**a**) Workflow used for the identification of cancer-related DNA variants from firefighters in the GFS cohort. The cfDNA was purified from blood collected from firefighters between February 2020 and April 2023. The cohort comprised firefighters who had attended the Grenfell Tower fire in 2017 and some who had not. cfDNA isolated from plasma was quantified using the Qubit fluorometric assay and further processed for qualitative analysis by next-generation sequencing (NGS). The numbers in parentheses indicate the number of firefighters in the individual subsets at each stage of the workflow. (**b**) Proportion of firefighters (n = 339) in each category of fire smoke exposure based on attendance at the Grenfell Tower fire (see Methods section). (**c**) Proportion of firefighters (n = 339) in each tertile of fire attendance on the basis of their historical smoke exposure (see “Methods” section). (**d**, **e**) Concentrations of cfDNA in the two subsets of firefighters and in the total cohort of firefighters (All). The median (red dots) and interquartile ranges are shown; the lower boundary indicates the 1st quartile (the 25th percentile), and the upper boundary indicates the 3rd quartile (the 75th percentile).

CfDNA is a promising noninvasive biomarker for detecting and monitoring cancer^23,24^. Studies have consistently shown that blood-derived plasma cfDNA levels are significantly elevated in cancer patients^23–26^. In this analysis, we isolated and analysed cfDNA obtained from 342 LFB firefighters. The concentration of isolated cfDNA was measured via a fluorometric assay (Qubit Fluorometric Quantitation; workflow shown in Fig. 1a). We evaluated whether exposure to Grenfell Tower fire smoke and/or high historical occupational smoke exposure (HSE, also see “Methods”) was associated with increased plasma levels of cfDNA. Our results revealed that the levels of cfDNA in plasma were similar in the firefighters in all of the GFS exposure categories (Fig. 1b,d) and in all of the HSE categories (Fig. 1c,e), suggesting that while plasma cfDNA can be effectively measured in our cohort, its levels do not vary as a result of fire smoke exposure.

### Optimising a targeted sequencing workflow for the identification of cancer-associated variants in isolated cfDNA

Analysis of cfDNA, with a focus on oncogenic mutations, can yield significant insights into the underlying biology of tumours^27,28^. Next-generation sequencing (NGS) of cfDNA makes it possible to detect somatic mutations in key cancer-related genes. The presence and allele frequency of mutations in cfDNA have been shown to correlate with tumour burden and can be used to help monitor treatment response and direct the treatment of minimal residual disease^27,29^.

**Fig. 2:**
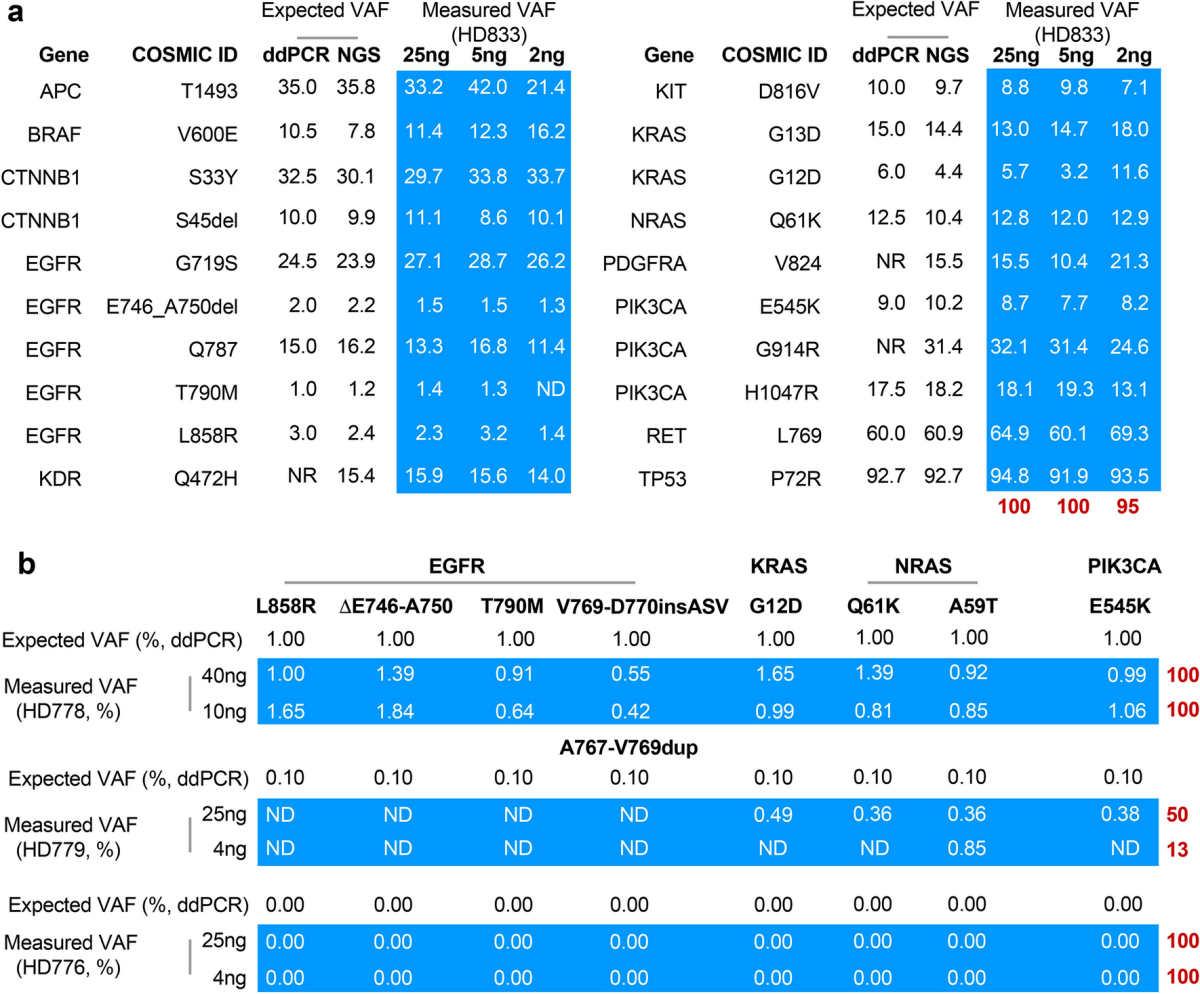
Validation of the assay using reference standards for NGS sequencing. (**a**) The sensitivity (red numbers) of the CHPv2 panel was assessed using three different amounts of the OncoSpan cfDNA standard (HD833). This standard includes both DNA variants with known COSMIC IDs and variants involving insertions or deletions (INDELs). The HD833 OncoSpan standard contains variants confirmed by ddPCR with orthogonal validation by next-generation sequencing (NGS). CHPv2 covers 20 variants in HD833. Our assay measured 20 variants (boxed in light blue) contained in this cfDNA standard. (**b**) The sensitivity and specificity of the CHPv2 panel were tested using the OncoSpan cfDNA standards with allele frequencies of 1% (HD778), 0.1% (HD779) or 0% (HD776) in EGFR, KRAS, NRAS and PIK3CA confirmed by ddPCR. NR, not reported; ND, not detected. The sensitivity of each analysis is shown in red. For the 0% control, this value depicts specificity, showing that no false-positives were detected. All the numerical values shown are percentages.

AmpliSeq for Illumina Cancer Hotspot Panel v2 (CHPv2 panel) is a targeted NGS assay that can identify somatic mutations in hotspot regions of 50 key cancer genes, as identified in the Catalogue of Somatic Mutations in Cancer (COSMIC). The panel comprises 207 amplicons covering approximately 2800 COSMIC mutations and can be used to detect somatic hotspot mutations in cancer-associated genes. In this study, the CHPv2 panel was used to assess the presence of somatic mutations in cancer genes in cfDNA in the GFS cohort. The CHPv2 panel has been optimised by the manufacturer for use in analysing DNA extracted from formalin-fixed, paraffin-embedded (FFPE) tissues at input quantities ranging from 1 to 100 ng. The sensitivity of the CHPv2 panel in detecting cancer-associated somatic mutations in cfDNA was assessed using a panel of commercial controls and different input quantities of four commercially available cfDNA standards to generate libraries for NGS. The HD833 cfDNA standard comprises 20 variants within the target region of the CHPv2 panel. Seventeen of these variants were confirmed by digital droplet PCR (ddPCR), and the remaining 3 variants were confirmed by NGS (Fig. 2a). When 5 ng or 25 ng of HD833 control input DNA was used, it was possible to detect true positive variants with a sensitivity of 100%, whereas when 2 ng of input DNA was used, the sensitivity was reduced to 95%.

To assess the ability of the CHPv2 panel to detect low-frequency variants, two standards, HD778 and HD779, were used. These standards harbour 8 pathogenic variants at variant allele frequencies of 1% and 0.1%, respectively. In the case of HD778, 100% sensitivity was achieved with both 10 ng and 40 ng of input DNA (Fig. 2b). However, the panel was unable to reliably detect variants that were present at a frequency of 0.1%, as demonstrated by the poor sensitivity achieved using this control at 4 ng and 25 ng of input DNA (Fig. 2b). To assess the specificity of the assay, the control standard HD776, which lacks the 8 pathogenic variants present in the HD778 and HD779 standards, was used. Our analysis of the HD776 control confirmed the absence of these 8 variants. Thus, our assay achieved a specificity of 100% for these variants (Fig. 2b).

**Fig. 3:**
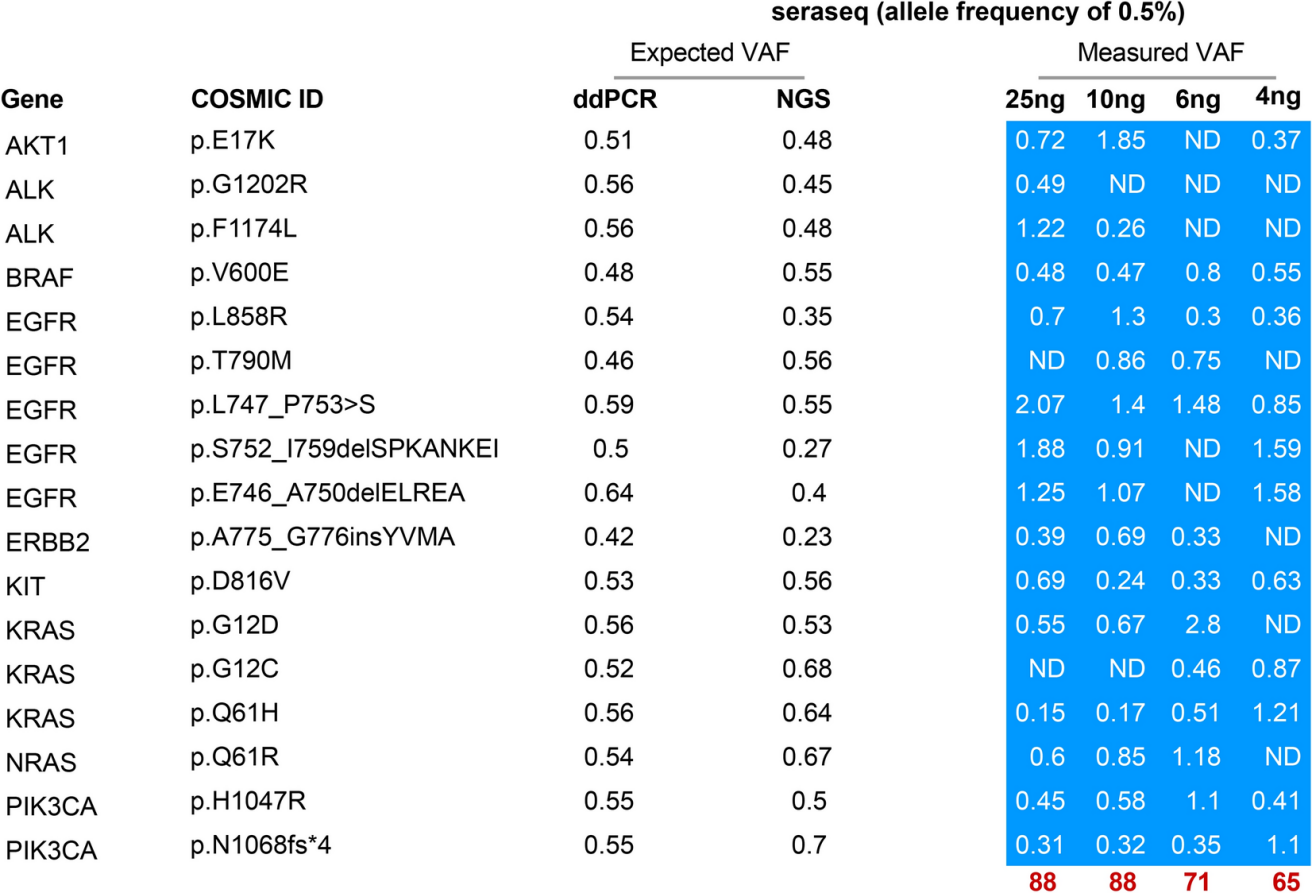
Assessment of the sensitivity of the assay through the use of a low-variant reference standard. The sensitivity (red numbers) of the CHPv2 panel when different amounts of input DNA were used was assessed using the Seraseq^®^ cfDNA Complete™ Reference Material AF0.5%. This standard includes both DNA variants with known COSMIC IDs and variants involving insertions or deletions (INDELS). The variants in this standard were confirmed by ddPCR with orthogonal validation by NGS, corresponding to the columns reporting the expected VAF. The measured VAF for each locus determined by NGS is shown boxed with a light blue background. ND, not detected.

The sensitivity of our NGS assay for detecting low-frequency variants in varying amounts of cfDNA was also tested. Using a cfDNA control with a variant allele frequency of 0.5% (Seraseq), we tested input amounts of cfDNA ranging from 4 to 25 ng. We found that when either 10 or 25 ng of input cfDNA was used, our assay achieved a sensitivity of 88%; however, the sensitivity of the assay decreased when 6 ng or 4 ng of input cfDNA was used (Fig. 3).

Taken together, the data show that the CHPv2 panel can be used to detect variants present at VAFs of 1% or greater in cfDNA using a minimum input amount of 5 ng cfDNA and an average read depth greater than 1000 reads.

### Targeted analysis of oncogenic variants in cfDNA in a subset of the Grenfell firefighters cohort

**Fig. 4:**
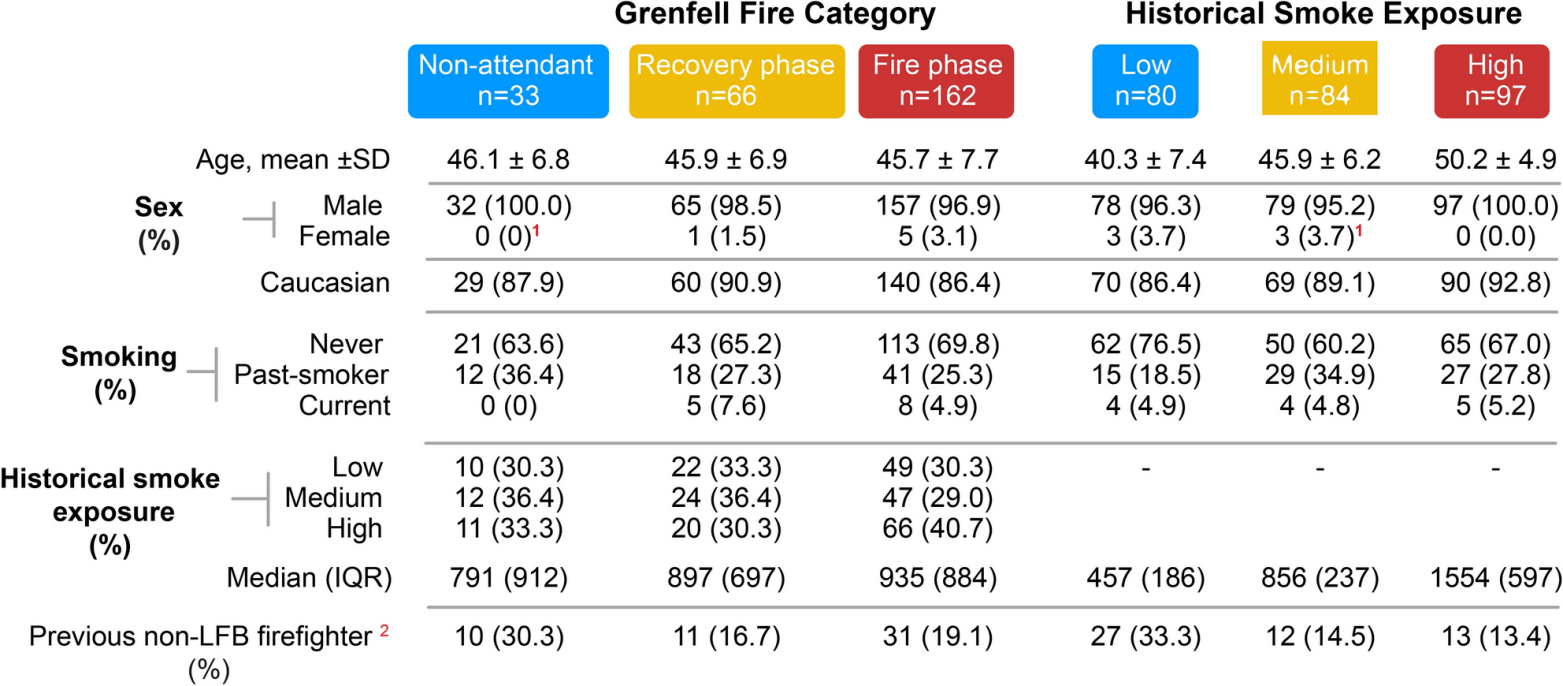
Demographics of the firefighters comprising the cohort in which NGS was performed. Descriptive statistics of the 261 firefighters whose cfDNA was analysed by NGS. IQR, interquartile range, is shown as the difference between quartile 3 and quartile 1. The values for the median and IQR are related to the number of fires attended by the firefighters. The data are presented as the number of firefighters, n (%), unless otherwise indicated. (^1^) One person did not declare their sex and therefore is not included in these numbers. (^2^) A “previous non-LFB firefighter” refers to a firefighter who participated in fires in addition to those recorded by the LFB.

The presence of oncogenic DNA variants in cfDNA isolated from the plasma of a subset of the GFS cohort was then assessed (Fig. 1a). We obtained sufficient cfDNA for qualitative analysis of DNA variants from 261 of the 342 samples quantified using our assay; further analysis was performed on these 261 samples. The demographics of this subset of the cohort (Fig. 4) were stratified by (i) their Grenfell Tower exposure and (ii) their historical occupational smoke exposure. The majority of the GFS participants were male (n = 254, 97.3%) and Caucasian (n = 229, 87.7%). The firefighters who attended the fire phase of the Grenfell Tower fire (the first 23 h of the fire) were of similar age to the firefighters who attended the recovery phase of that fire (the subsequent 13 days) and those who did not attend the fire at all (‘nonattendees’). With respect to the firefighters’ historical occupational fire smoke exposure, those in the highest tertile of smoke exposure (n = 97) were, on average, 10 years older than those in the lowest tertile of fire smoke exposure (50.2 years vs. 40.3 years). The median numbers of fires attended by the nonattendee, recovery phase and fire phase firefighters in their lifetimes were 791, 897 and 935, respectively.

**Fig. 5:**
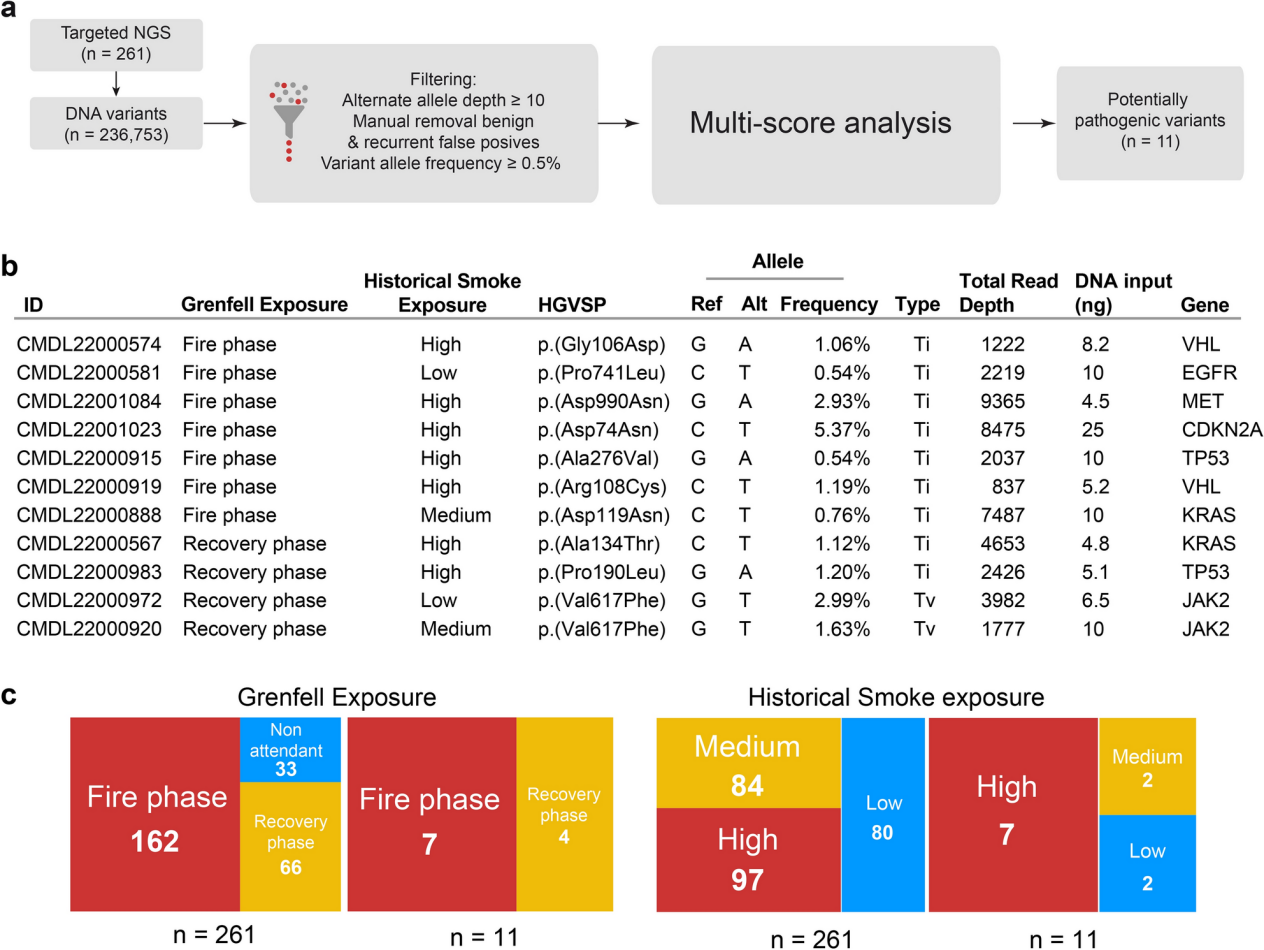
Analysis of cancer-associated DNA variants in Grenfell Fire firefighters. (**a**) Workflow used in the identification of somatic variants in the cfDNA of LFB firefighters in the GFS. Three different filtering criteria were used, followed by a multiscore analysis of the filtered variants. We used five independent scores (multiscore analysis) to classify these variants as pathogenic (see Methods section). (**b**) List of individual cfDNA variants present in the GFS cohort. HGVSP, Human Genome Variation Society amino acid ID; Ref, reference allele; Alt, alternate allele. “Type” indicates whether the variant corresponds to a transition (Ti) or a transversion (Tv) event. (**c**) Exposure categories within the cohort of firefighters analysed by NGS (n = 261) and for the firefighters in whom pathogenic variants were detected (n = 11). The numbers inside the boxes indicate the number of observations per category.

The CHPv2 panel was used to test for the presence of cancer-associated somatic mutations in cfDNA extracted from plasma in the 261 members of the GFS cohort. We used a series of criteria to filter out all known benign variants, sequencing artefacts and germline variants (Fig. 5a and “Methods”). The variants were defined as pathogenic based on the criteria provided by the Association for Molecular Pathology and the College of American Pathologists^30^.

Eleven pathogenic variants were detected in 11 firefighters (4.2% of the firefighters whose cfDNA was tested for the presence of pathogenic variants) (Fig. 5b, c and Supplementary Table 4). Seven of these were found in firefighters who attended the fire phase of the Grenfell Tower fire (7/162, 4.3% of the firefighters in this group; 95% CI 2.1–8.6). We also found four pathogenic variants in the 66 firefighters who attended the recovery phase of that fire (4/66, 6.1%, 95% CI 2.4–14.6). No variants were detected in any of the 33 firefighters who did not attend the Grenfell Tower fire (0%, 95% CI 0–10.4) (*P* = 0.49; Fisher’s exact test overall, Fig. 5).

Among the firefighters in the high, medium and low tertiles of historical occupational smoke exposure, pathogenic variants were found in 7.2% [95% CI 3.5–14.2] (7/97), 2.4% [95% CI 0.7–8.3] (2/83) and 2.5% [95% CI 0.7–8.6] (2/81), respectively (*P* = 0.23; Fisher’s exact test overall). All firefighters identified as having a pathogenic variant were male, and the mean age of these firefighters was 48.5 years (95% CI 45.4–51.6), whereas those in whom no pathogenic variant was found had a mean age of 45.6 years (95% CI 44.7–46.6). The firefighters who had pathogenic variants had a lower prevalence of current/previous smoking than those who did not (18.2% vs. 32.2%). They also had attended a greater number of fires [median (IQR) 1256 (826) vs. 892 (857)]. The characteristics of firefighters whose plasma cfDNA was sequenced (261/685) were compared with those of firefighters whose plasma cfDNA was not sequenced (424/685) (Supplementary Table 1) but who were similar in age, sex, ethnicity, smoking status and cumulative smoke exposure. However, of the 261 firefighters whose cfDNA was sequenced, a slightly higher percentage (62.1%) had attended the fire phase of the Grenfell Tower fire than the corresponding percentage (48.1%) of the 424 firefighters whose cfDNA was not sequenced.

The seven variants observed in the fire phase group comprised a variant in *VHL* (G106D) found in small cell carcinoma of the lung; a variant in *EGFR* (P741L), found in different solid cancers such as astrocytomas, lung, skin and soft tissues; a variant in *MET* (D990N) found in skin and lung cancer; a variant in *CDKN2A* (D74N), found in different solid cancers, such as breast, lung, oesophagus and skin; a variant in *TP53* (A276V), found in different solid cancers such as breast, pancreas, liver, large intestine and central nervous system; a variant in *VHL* (R108C), found in kidney cancer and a variant in *KRAS* (D119N), found in skin cancer, cervix cancer and haematopoietic and lymphoid cancers.

The three variants observed in the recovery phase group comprised a variant in *KRAS* (A134T), found in low grade mucinous adenocarcinoma and brown tumour of the jaw; a variant in *TP53* (P190L), found in multiple solid cancers such as those in the large intestine, oesophagus, lung and liver and haematopoietic and lymphoid cancers and a variant in *JAK2* (V617F), found in myeloproliferative neoplasms. In conclusion, we identified only 11 pathogenic DNA variants in the subsample of the GFS cohort.

## Discussion

We sequenced cfDNA extracted from the blood of 261 LFB firefighters and detected 11 pathogenic variants. No firefighter exhibited more than one variant. All of the variants were found in firefighters who had attended the Grenfell Tower fire during either the fire phase or the recovery phase; however, the plasma cfDNA of only a very small sample of non attendees (n = 33) was analysed by NGS. A slightly greater proportion of pathogenic variants was observed in the firefighters who were in the highest tertile of historical occupational fire smoke exposure; however, there was no evidence for a significant difference in the number of pathogenic variants in the groups with different historical fire smoke exposures or in the groups whose members were exposed to different phases of the Grenfell Tower fire. Given the correlation between age and cancer incidence, it is possible that the greater presence of harmful variants in the highest tertile of historical occupational fire smoke exposure is linked to the increased age of these individuals, as those in the highest tertile were, on average, 10 years older than those in the lowest tertile. Our analysis was exploratory, and we were not able to establish a causal link between historical occupational smoke exposure or attendance at the Grenfell Tower fire and the presence of pathogenic variants in these firefighters. Due to the small number of pathogenic variants identified, we did not adjust for any potential confounders, however, a similar prevalence of never smokers was reported across Grenfell exposure groups and it is unlikely other lifestyle factors would have been associated with the exposure categories.

The risk of cancer in firefighters varies by country due to differences in the practices used, including the type and use of protective equipment and decontamination protocols and whether the firefighters are primarily exposed to urban fires, to wildfires or to both types of fires. A recent meta-analysis of 24 studies of firefighters revealed an increase in cancer incidence in firefighters in the USA; outside the USA, an increase in the risk for Hodgkin’s disease and malignant melanoma was reported^31^. A limited number of studies of UK firefighters and cancer have been published. One study that used data collected between 1965 and 1993 reported reduced cancer incidence and mortality in a group of more than 5000 male firefighters^32^. A retrospective review of medical service records of more than 2200 serving Scottish male firefighters revealed that the overall mean cancer incidence and the mortality rate due to cancer in these firefighters were lower than those in age-matched male controls from the general population^33^. However, that study did report an increase in the incidence of melanoma and kidney cancer and in mortality from kidney cancer^33^.

In contrast, a very recent study in which publicly available cancer mortality rates in 672 male firefighters in Scotland were compared with those of the general male Scottish population reported an elevated standardised mortality ratio of 2.71 (1.71–4.00) due to malignant neoplasms^34^. However, the authors were unable to adjust for possible confounders, including cigarette smoking, alcohol consumption and comorbidities. Another complicating factor is that firefighters work shift patterns and may also differ from nonfirefighters in other ways; this may impact their cancer risk and would provide alternative explanations for the findings of this study.

Firefighting has traditionally been a male-dominated profession, and this was reflected in the very small number of female firefighters included in our study. We acknowledge that this limits our ability to draw conclusions about the cancer risks in females.

Our study was not designed to evaluate the association between being a firefighter and the risk of cancer, as we included only firefighters; therefore, we are unable to determine whether equivalent prevalences of pathogenic variants exist in populations that have not been exposed to fire smoke. There is significant interest in developing blood tests that can be used as screening tools for the early detection of an array of different known cancers in asymptomatic individuals. The possible targets include circulating tumour DNA, microRNAs and circulating tumour cells, and research in which these potential targets are being evaluated in real-world populations is underway^35^. In the United States, some firefighters are offered screening for cancer using a panel of protein cancer biomarkers (https://lp.onetestforcancer.com), but no such programme exists in the UK.

We detected variants at low variant allele frequencies (0.5–5.5% VAFs) in 11 subjects in our cohort. Although these variants have been detected in individuals with cancers of different types (Supplementary Table 4), the clinical significance of our results cannot be determined by this assay. The results only indicate that it is possible to detect low-level somatic pathogenic variants that are present in cfDNA. Unlike a recent study in which cfDNA from 55 healthy individuals was analysed^36^, we did not detect any known pathogenic cancer disposition germline variants in our cohort.

The majority of the 11 DNA variants identified were transitions (C to T or G to A) rather than transversions (C to A or G to T). Transition variants are more common than transversion variants; they result in relatively minor structural changes in DNA and, in general, are less likely to result in serious disease. However, oxidative stress caused by exposure to environmental toxins can cause specific types of DNA damage and influence the mutational landscape^37,38^. Exposure of this type may preferentially induce transitions that alter C and G bases while leaving T and A bases relatively unchanged. Alternatively, our sequencing methodology may have biases that affect allele detection; for example, certain sequencing technologies may have varying sensitivities to nucleotide changes of different types, and this could potentially lead to missed reference alleles containing T or A, even if no variant is identified more than once.

At present, there are no guidelines or procedures for the use of cfDNA testing to detect pathogenic variants in healthy populations or for its use in the early detection of cancer. To determine whether some or all the variants detected by our assay have clinical significance, longitudinal screening can be performed to monitor increases in clone size (VAF) and to monitor whether the subject develops any type of cancer. The main conclusion of this study is that there is no association between fire smoke exposure and the pathogenic variants that were detected in the plasma of the subjects tested.

This study used the largest cohort of UK firefighters who have been recruited to date for a follow-up study and for whom there is historical fire attendance data to allow for detection of a possible dose‒response relationship between exposure and outcomes. We were able to contact firefighters who had left LFB since 2017 to mitigate some of the healthy worker effects inherent to occupational studies. Funding constraints, however, did not allow us to analyse the complete GFS cohort, and the final sample analysed included only a small number of female firefighters. Consistent with previous recommendations^39–41^, we did not perform a post hoc power analysis as part of our interpretation of the findings. Large confidence intervals were obtained for the estimated proportions in this study; a larger future study would likely yield more accurate estimates of the prevalence of variants in each exposure group.

We were not able to account for fire attendance external to the LFB, and this may have resulted in an underestimation of the total number of fires attended. However, only 20% of the firefighters stated that they had worked for a fire and rescue service other than LFB, and at least some of this work is likely to have involved being a retained (volunteer) firefighter who received relatively little additional fire smoke exposure; therefore, this is unlikely to have had a significant impact on our findings.

Whilst it would have been ideal to obtain fire attendance records for those working for services other than LFB, this would have involved requesting historical, individual-level data from around 30 different fire services. In addition, data records pre-2000 is likely to be non-electronic and so records may not have been available or easy to compile. We were not able to account for absence due to sickness prior to 2009, and this may have led to an overestimation of the number of fires attended by those employed at the LFB during this period. The fires recorded after 2009 included all types of fires, whereas only primary and secondary fire data were available for the period prior to 2009. This would be expected to have had a negligible effect on the results, as LFB records show that primary and secondary fires account for more than 99% of fires attended^42^.

The NGS panel used in this study was tailored to detect hotspot mutations in 50 genes that are known to be associated with cancers, including lung, colorectal, melanoma, prostate and other solid cancers (see “Methods” for details). Notably, the absence of pathogenic variants in these genes in the studied samples does not exclude the possibility that pathogenic variants are present in other cancer-related genes or in regions of the target genes that were not examined. For example, the panel does not include genes involved in DNA repair; mutations in those genes can play a role in prostate and testicular cancers, both of which are associated with occupational exposure in firefighters^43^. Improving the depth of future investigations could involve implementing a larger panel of genes frequently mutated in cancer with all exons targeted rather than just the hotspot regions in a bigger cohort of samples. In addition, incorporating molecular barcodes in the library preparation prior to sequencing would further enhance the quality of data obtained and improve the limit of detection. Whilst whole genome sequencing would provide the most comprehensive analysis; this was not feasible due to limited availability of blood samples for analysis. Furthermore, it would be cost-prohibitive to carry out high sequencing depth to detect variants at low variant allele frequencies on such a large number of samples.

In conclusion, this study lays the foundation for future research of cancer-related molecular markers in firefighters who have fire smoke exposure in urban areas. More research is needed to understand their risks of developing cancer and how this might be prevented.

## Methods

### Organising a cohort of LFB firefighters

More than 1000 firefighters from the LFB attended the fire at Grenfell Tower in June 2017. We established a cohort of firefighters who were employed by LFB at that time, many of whom attended the Grenfell Tower fire (see below), and used this cohort to longitudinally investigate a wide range of potentially harmful consequences, including cancer, that may result from exposure to smoke from urban fires. Operational firefighters employed by LFB in June 2017 were invited to attend a study visit and recruited for a cohort study between 27/07/2020 and 19/04/2023. After informed consent was obtained, each firefighter completed a questionnaire that included questions about demographics, medical history and historical occupational fire smoke exposure, including the firefighter’s attendance, and, if relevant, his or her role at the Grenfell Tower fire in 2017. A total of 685 firefighters were recruited for the cohort study. Blood samples were obtained from 679 of these firefighters and processed on the same day as the study visit. cfDNA was isolated from the blood samples and stored for later quantitative and qualitative analysis (Fig. 1). Blood samples for the cfDNA analysis were collected from the first 352 participants who had entered the study by February 2022 as the study was ongoing.

### Declarations

Approval for the Grenfell Firefighters Study was granted by the North West London Ethics Committee (19/LO/1847). All methods were carried out in accordance with relevant guidelines and regulations. Participants were informed about the study and provided informed consent.

### Exposure categories

Full occupational histories related to the number of fires attended while working for LFB were obtained from the fire service records. Two sets of exposure categories were developed from the questionnaire data and lifetime occupational fire attendances. The first exposure classification was based on the firefighter’s role in the Grenfell Tower fire, at which some firefighters experienced unusually high exposure to fire smoke. Three categories within this classification were defined, as follows: (1) attended during the fire phase (the first 23 h), (2) attended during the recovery phase only (between 23 h and 13 days after the fire), and (3) did not attend the fire (‘nonattendees’). The second exposure classification was based on occupational lifetime fire attendance using tertiles of number of fires attended: (1) high, (2) medium, and (3) low. Individual-level data on the number of fires attended were available from 2009 onwards; for the years prior to 2009, an estimate of the number of fires attended by each individual was made using station-level data after standardised adjustments for shift work and annual leave (Supplementary Table 2). It was not possible to account for sickness absence prior to 2009, but it was accounted for from 2009 onwards.

### Blood collection

Blood (20 ml) was obtained from 679 participants by venipuncture and collected into K_2_ EDTA-containing collection tubes (BD Biosciences, San Jose, CA, USA). The collected blood was centrifuged at 1000 × g for 10 min, and the supernatant of this centrifugation (the plasma) was centrifuged at 1000 × g for an additional 10 min. The blood samples were processed promptly, within 15 min of being taken, to reduce haemolysis; samples that showed evidence of haemolysis on visual inspection were excluded from further analysis.

### Extraction and quantitation of DNA

cfDNA was extracted from 1-ml plasma samples using a QIAsymphony DSP Circulating DNA Kit (Cat. No. ID: 937556; Qiagen) and eluted in 60 μl of the elution buffer (10 mM Tris–Cl (pH 8.3), 0.1 mM EDTA and 0.04% NaN_3_) according to the protocol provided with the kit. The purified DNA was quantified using a Qubit dsDNA High Sensitivity Kit with a detection range of ≥ 10 pg/μl (Thermo Fisher Scientific, Cat. No. Q33230); an aliquot of the purified DNA was also analysed on Cell-free DNA ScreenTape (Agilent). The extracted DNA was stored at − 80 °C until use.

### Library generation

Libraries were prepared from various amounts of cfDNA using AmpliSeq for Illumina Cancer Hotspot v2 Panel (Cat. No. 20019161) and AmpliSeq™ Library PLUS Kit for Illumina (Cat. No. 20019103) according to the manufacturer’s directions. This panel allows the detection of pathogenic DNA variants associated with multiple types of cancer, including bladder, lung, colon, breast, and prostate cancer, as well as melanoma. It allows the analysis of cancer-associated hotspot variants in the following genes: *ABL1*, *EGFR*, *GNAS*, *KRAS*, *PTPN11*, *AKT1*, *ERBB2*, *GNAQ*, *MET*, *RB1*, *ALK*, *ERBB4*, *HNF1A*, *MLH1*, *RET*, *APC*, *EZH2*, *HRAS*, *MPL*, *SMAD4*, *ATM*, *FBXW7*, *IDH1*, *NOTCH1*, *SMARCB1*, *BRAF*, *FGFR1*, *JAK2*, *NPM1*, *SMO*, *CDH1*, *FGFR2*, *JAK3*, *NRAS*, *SRC*, *CDKN2A*, *FGFR3*, *IDH2*, *PDGFRA*, *STK11*, *CSF1R*, *FLT3*, *KDR*, *PIK3CA*, *TP53*, *CTNNB1*, *GNA11*, *KIT*, *PTEN*, and *VHL*.

### Targeted next-generation sequencing

Next-generation paired-end (2 × 150 bp) sequencing of pooled libraries (approx. 80/pool) was performed on a NextSeq 2000 platform (Illumina) using NextSeq 2000 P1 Reagents (300 cycles). The sequencing data were aligned to the human genome version hg19 (Grch37) and analysed using Illumina’s DNA Amplicon app v2.1.1. Before analysing the DNA isolated from plasma samples, we assessed the sensitivity and specificity of the assay using commercial cfDNA standards (Oncospan, Cat. Nos. HD833, HD778, HD779, HD776 and Seraseq® cDNA Complete Mutation Mix AF0.5%) that contain variants in several key oncogenes and tumour suppressor genes at known variant allele frequencies (VAFs)). Sequencing depth, also known as read depth or depth of coverage, refers to the number of times a specific base (nucleotide) in the DNA is read during the sequencing process. It reports the average number of times a given position in the genome is sequenced. Greater sequencing depth increases confidence in the accuracy of the base calls at that position and helps reduce sequencing errors and noise. The mean read depths obtained for the samples in this study are reported in Supplementary Table 3.

### Variant filtering and classification

All the variants detected were manually confirmed across all the samples using Integrated Genomics Viewer 2.17. The variant allele frequency (VAF) was calculated as the proportion of total reads at a site containing the variant allele. Variants were filtered by first removing all variants present at VAF > 1% in population databases (GnomAD, ExAC), then by removing SNPs that are classified as benign or likely benign in the Clinvar database, then by removing sequencing artefacts that were recurrent in all samples, and finally by removing variants with VAFs > 30%, which are considered likely germline SNPs. Multiscore analysis of pathogenic variants involved filtering the data frame in R using the following expression in the dplyr package^44^: filter((Polyphen =  = “damaging” | Polyphen =  = “probably damaging”) & COSMIC_ID =  = TRUE & Sift_score =  = “deleterious” & Revel_score >  = 0.6 & Frameshift =  = TRUE). For samples with < 10 ng of input DNA, the minimum VAF for reporting pathogenic variants was 1%. For all other samples, we used a minimum VAF cutoff of 0.5%. Variants were further classified based on published criteria^30,45^. Variants that did not meet the filtering criteria are not reported in our study for reasons related to the anonymity and traceability of the individual firefighters who agreed to participate in this study.

### Statistical analysis

Statistical analyses were performed using R version 4.4.1. Data are presented as the mean (SD) and median (IQR) as appropriate or n (%). The proportions of pathogenic variants were compared across the HSE and GFS exposure categories using the Fisher’s exact test. Confidence intervals for the proportions were also calculated using the Wilson interval for small samples.

## Supplementary Information

Supplementary Information.

## Data Availability

All the data generated or analysed during this study are included in this article and are available upon reasonable request to the corresponding authors, Johanna Feary or L. Miguel Martins.

## Abbreviations

cfDNA: Circulating free DNA
PCR: Polymerase chain reaction
NGS: Next-generation sequencing
ddPCR: Droplet digital PCR
VAF: Variant allele frequency
SNPs: Single nucleotide polymorphisms
SNVs: Single nucleotide variants
INDELs: Insertions or deletions
CHPv2: AmpliSeq for Illumina Cancer Hotspot Panel v2
FFPE: Formalin-fixed paraffin-embedded
