# Supplementary material for: Assessment of cancer biomarkers in the Grenfell firefighter cohort study
Source: Sci Rep. 2025 May 7;15:15784. doi: 10.1038/s41598-025-95991-y (PMC12059041; doi:10.1038/s41598-025-95991-y)
Supplement: Supplementary file 1 — Supplementary Information. [file 41598_2025_95991_MOESM1_ESM.docx]

## Feary et. al

## SUPPLEMENTARY DATA

|  | | **Sequenced** | |
| --- | --- | --- | --- |
|  |  | No  n=424 | Yes  n=261 |
|  | Age, mean ± SD | 45.0 ± 7.0 | 45.0 ± 7.0 |
| Sex^1^ number (%) | Male | 396 (93.4) | 254 (97.7) |
|  | Female | 28 (6.6) | 6 (2.3) |
|  | Caucasian (%) | 378 (89.2) | 229 (87.7) |
| Smoking number (%) | Never | 289 (68.2) | 177 (67.8) |
|  | Past-smoker | 102 (24.1) | 71 (27.2) |
|  | Current | 33 (7.8) | 13 (5.0) |
| Grenfell Exposure number (%) | Non-attendees | 101 (23.8) | 33 (12.6) |
|  | Recovery | 119 (28.1) | 66 (25.3) |
|  | Fire | 204 (48.1) | 162 (62.1) |
|  | Historical Smoke Exposure, median (IQR) | 831 (713) | 916 (849) |

## Supplementary Table 1. Comparison of the participant blood samples subjected to NGS analysis with those that were not. Data are presented as n (%) unless otherwise stated. (1) One person did not declare their sex and therefore is not included in these numbers.

|  | Period | |
| --- | --- | --- |
|  | From 2009 onwards | Up to 2009 |
| **Data collection** | Individual level | Number of fires estimated using manually recorded station worked at from individual employment records^1^ |
| **Leave management** | Accounted in individual records | Sick leave not accounted for Annual leave - made a 11% reduction^2^ |
| **Type of fire** | All types | Primary and secondary |

## Supplementary Table 2. Summary of the available data on historical fire smoke exposure.

Individual-level data on the number of fires attended were available from 2009 onwards. For the years preceding 2009 (1), we manually extracted data from employment records of the fire station at which each individual had worked. We then estimated the number of fires each individual would have attended each year on the basis of national data on the number of fires attended by the employees at each fire station between 2000 and 2013 and divided this number by 4 to account for the 4 ‘watches’ or teams at each station. We were able to account for the number of months the firefighters had worked at each station, and we included a standard reduction for annual leave. We were not able to account for sick leave or for fires classified as “nonprimary” or “secondary” (for example, chimney fires) prior to 2009. (2) An 11% reduction corresponds to a calculation that considers the annual leave for that period.

| **Supplier** | **Standard reference** | **DNA amount (ng)** | **Amplicon mean read depth** |
| --- | --- | --- | --- |
| Horizon | HD833 | 25 | 7495 |
|  | HD833 | 5 | 3357 |
|  | HD833 | 2 | 4530 |
|  | HD786 | 25 | 9519 |
|  | HD778 | 10 | 3154 |
|  | HD778 | 40 | 1690 |
|  | HD776 | 25 | 2630 |
|  | HD776 | 4 | 2085 |
|  | HD779 | 25 | 2630 |
|  | HD779 | 4 | 2378 |
| SeraCare | Seraseq® cDNA Complete Mutation Mix AF0.5% | 4 | 2468 |
|  | Seraseq® cDNA Complete Mutation Mix AF0.5% | 6 | 4086 |
|  | Seraseq® cDNA Complete Mutation Mix AF0.5% | 10 | 3700 |
|  | Seraseq® cDNA Complete Mutation Mix AF0.5% | 25 | 2418 |

## Supplementary Table 3. Mean read depths obtained for the commercial cfDNA standards used in this study.

Average mean depth of the 207 amplicons obtained using different amounts of the commercial cfDNA standards used in this study.

| **Gene** | **Variant** | **Cancer type where the variant has been detected** | **Source database or notes** | **References** |
| --- | --- | --- | --- | --- |
| VHL | G106D | Small cell carcinoma of the lung | Non-truncating non-synonymous variant is located in a mutational hot spot and/or critical and well-established functional domain, extremely low frequency in population databases. | https://www.oncokb.org/gene/VHL/G106; https://cancer.sanger.ac.uk/cosmic/mutation/overview?id=104517422 |
| EGFR | P741L | Different solid cancers e.g. astrocytomas, lung, skin and soft tissues | https://cancer.sanger.ac.uk/cosmic/mutation/overview?id=103095913 | PMID:21771097; https://www.oncokb.org/gene/EGFR/P741L |
| MET | D990N | Melanoma, lung | https://cancer.sanger.ac.uk/cosmic/mutation/overview?id=189384257 | PMID:25453846; PMID:25314153; PMID:25142162 |
| CDKN2A | D74N | Different solid cancers e.g. breast, lung, oesophagus, melanoma and thyroid | https://www.oncokb.org/gene/CDKN2A/D74N | https://cancer.sanger.ac.uk/cosmic/mutation/overview?id=147104192 |
| TP53 | A276V | Different solid cancers e.g. breast, pancreas, liver, large intestine and central nervous system | Functional data suggests that the protein is only partially functional as a result of the mutation | https://cancer.sanger.ac.uk/cosmic/mutation/overview?id=104063475; https://tp53.cancer.gov/results_gene_mut/gv |
| VHL | R108C | Renal cell carcinomas | Present in mutational hotspot and *in silico* algorithms consider this change as deleterious | https://pubmed.ncbi.nlm.nih.gov/38969834/; https://pubmed.ncbi.nlm.nih.gov/29219616/ |
| KRAS | D119N | Different cancers e.g. skin, cervix, haematopoietic and lymphoid | https://www.oncokb.org/gene/KRAS/D119N | https://cancer.sanger.ac.uk/cosmic/mutation/overview?id=104003896 |
| KRAS | A134T | Low grade mucinous adenocarcinoma and brown tumour of jaws | Absent from population databases. *In silico* algorithms consider this change as deleterious |  |
| TP53 | P190L | Multiple cancer types e.g. large intestine, haematopoietic, lymphoid, oesophagus, lung and liver | Published functional studies demonstrate a damaging effect: loss of growth suppression activity, partially functional transactivation, and a dominant-negative effect (Kato et al., 2003; Jordan et al., 2010; Giacomelli et al., 2018; Kotler et al., 2018); Not observed at significant frequency in large population cohorts (gnomAD); *In silico* analysis supports that this missense variant has a deleterious effect on protein structure/function. | This variant is associated with the following publications: (PMID: 12124823, 15510160, 20407015, 32817165, 34863587, 32658383, 35974385, 30224644, 29979965, 12826609); https://cancer.sanger.ac.uk/cosmic/mutation/overview?id=103965948 |
| JAK2 | V617F | Myeloproliferative neoplasms | https://www.oncokb.org/gene/JAK2/V617F | https://www.ncbi.nlm.nih.gov/pubmed/15781101; https://www.ncbi.nlm.nih.gov/pubmed/15837627; https://www.ncbi.nlm.nih.gov/pubmed/15863514 |

## Supplementary Table 4. Available information on the cancer-associated pathogenic DNA variants found in the Grenfell firefighters.

Information on the individual pathogenic cfDNA variants present in the GFS cohort is shown.
